# Treatment of Thoracolumbar Spinal Infections through Anterolateral Approaches Using Expandable Titanium Mesh Cage for Spine Reconstruction

**DOI:** 10.1100/2012/545293

**Published:** 2012-10-23

**Authors:** Tarantino Roberto, Marruzzo Daniele, Cappelletti Martina, De Giacomo Tiziano, Delfini Roberto

**Affiliations:** ^1^Neurosurgery, Department of Neurological and Psychiatric Sciences, Sapienza University of Rome, Rome, Italy; ^2^Department of “P. Stefanini” of General Surgery and Organs Transplant, Sapienza University of Rome, Rome, Italy

## Abstract

Pyogenic vertebral osteomyelitis (PVO) is still a rare pathology. However, its incidence is on the rise. This is due to an increasing population with predisposing factors. Also, the availability of more effective diagnostic tools has brought it increasingly to the surgeon's attention. In this study the patients were treated in the Neurosurgery Division of the Department of Neurological Sciences and Psychiatry of the Sapienza University of Rome, between 2001 and 2009. They had thoracolumbar pyogenic spondylitis. This study was undertaken in order to identify the correct diagnostic and therapeutic treatment needed in such cases. From the cases studied here, it is evident that spinal infections can be extremely insidious and that diagnosis tends to be reached late. Surgery, along with the antibiotic treatment, allows for eradication of the causes of the pathology by the reclamation of the affected region. Surgery is also fundamental in helping to recover vital functions and in restoring as much as possible the correct curvature of the rachises. The use of an anterolateral approach is dictated by the necessity of obtaining 360° stability as well as by the need to clear away extensive infections, which are not always reachable using a posterior approach.

## 1. Introduction

Spinal infections are quite rare pathologies, that is, 2% to 7% of all osteomyelitis [1]. Nevertheless predisposing factors such as diabetes, immune-depression, and advanced age can favour their appearance. 

 These infections are usually caused by saprophytes which are normally present in human organisms. In certain conditions they can become pathogens [2]. One germ in particular must always be taken into consideration in a differential diagnosis and that is the BK. Its frequency is on the increase due to the migration of people from developing to industrialised countries [3]. 

The diagnosis of spinal infection occurs late as these infections arise insidiously generally with the appearance of localised pain which gradually worsens. When there is radicular or spinal cord compression, the infection has already reached an advanced stage. The patient usually comes to the surgeon's attention once medical therapy has failed and thus when the infection is fairly advanced [4].

The treatment of the spondylodiscitis is essentially medical. However, when the infection fails to decrease or it progresses, intervention becomes necessary. Once neurological defects become apparent surgery is called for.

There are no specific guidelines on what would be the most appropriate surgical treatment [5].

Therefore, these cases were reexamined retrospectively in order to evaluate the clinical and medical aspects to take into consideration when deciding on the appropriate therapy for patients affected by spinal infections.

## 2. Materials and Methods

This study is based on the review of 16 clinical charts of patients affected by thoracolumbar pyogenic (tuberculotic and nontuberculotic) spondylitis (Table 1). They were treated surgically from 2002 to 2009 in the Neurosurgery Division of the Department of Neurological Sciences and Psychiatry of the University “La Sapienza”, Rome. Fourteen patients were operated on using an anterolateral and posterior column approach with the placement of an expandable titanium mesh (ETM) and pedicle screws. Two of these patients were treated using solely the anterolateral approach. None of the patients had had any previous operations on their spinal columns.

Prior to the operation, the patients were questioned as regards the duration of symptoms with especial attention paid to back pain, radicular pain, and paraesthesia. They then submitted to a neurological exam to evaluate their tendon reflexes, any signs of radicular or spinal cord compression, and the presence of neurological sphincter defects, sensitive, and/or motor impairment.

 The patients were evaluated in the preoperative and in postoperative course with the Frankel scale: *Grade A*: complete neurological injury—no motor or sensory function clinically detected below the level of the injury. *Grade B*: preserved sensation only—no motor function clinically detected below the level of the injury; sensory function remains below the level of the injury but may include only partial function (sacral sparing qualifies as preserved sensation). *Grade C*: preserved motor non-functional—some motor function observed below the level of the injury, but is of no practical use to the patient. *Grade D*: preserved motor function—useful motor function below the level of the injury; patient can move lower limbs and walk with or without aid, but does not have a normal gait or strength in all motor groups. *Grade E*: normal motor—no clinically detected abnormality in motor or sensory function with normal sphincter function; abnormal reflexes and subjective sensory abnormalities may be present.

The laboratory exams included a complete blood cell count with differential, erythrocyte sedimentation rate (ESR) and C-reactive protein (CRP) evaluation, in addition to peripheral blood cultures.

In three cases a CT guided biopsy with a needle via the pedicle was undertaken (in only one case a germ was isolated). All patients before the operation underwent an MRI in the T1 sequence with and without contrast, T2 and STIR, CT and a standard X-ray, this was to evaluate the extent of the infection, the possible compression of nerve structures, and the degree of bone erosion and kyphosis.

In selected cases a preoperative angiography with identification of the artery of Adamkiewicz was done. 

During the operation samples were taken for histological exams and cultures.

Following the operation antibiotic treatment was administered intravenously for four weeks and after that it was administered orally.

In the first week following the operation, a CT was performed to check the screw fixation system. Then, during followup, the patient was X-rayed in an upright position so as to control the reduction of the preop kyphosis and the possible shift or movement of the ETM or the positioning screws.

The Laboratory exams included a complete blood count, erythrocyte sedimentation rate (ESR), and C-reactive protein (CRP). These were evaluated to check on the infection and to determine the end of the antibiotic therapy.

The follow-up period ranged from 18 months to 4 years (mean: 32.8 months).

## 3. Results

The data obtained from the 16 cases is resumed in Table 1. The average age of the patients was 56.5 (range 21 to 80 years old), 10 patients (62.5%) were male and 6 (37.5%) were female. Fourteen patients (87.5%) had other pathologies. Eight patients (50%) came from non-European countries.

All presented with back pain or lumbar pain associated with radicular pain. In 12 patients, 75% of the cases, there were also neurological defects.

In all the patients the ESR and CRP were above the normal range, in 8 cases the white blood cell count was elevated.

The average duration of symptoms prior to surgical intervention was of 8 weeks (range 1 to 16 weeks). 

Fourteen out of 16 patients (87.5%) noted an improvement in their pain symptomology following the operation. Nine out of 16 patients who had preoperative neurological defects presented clinical improvement following the operation, 4 patients who had no neurological disorders remained free postoperatively, and 3 patients who had moderate or severe neurological disorders (Frankel A and C) did not present clinical improvement in the postoperative course.

In 3 cases, an intravascular aortic prosthesis was placed following a ruptured aneurism.

Fourteen of the cases had come to the neurosurgery department after having previously visited other specialists and having followed a course of antibiotics for a number of weeks.

In 7 cases the cultures were negative, in 6 cases the germ identified belonged to the staphylococcus family while in 3 cases it was the bacillus of Koch. In 12 cases the cultures obtained during the operation were negative and the germ responsible was isolated following a further cultural exam.

Out of the 12 patients who presented involvement of the dorsal rachises, 8 on the radiography exams showed a kyphosis of the spinal tract affected by the infection (range from 38° to 45°). Among the 4 patients with an involvement of the lumbar rachises, in one case the radiography showed a 62° increased lordosis, while for the rest of the patients there was a significant decrease in lordosis (range from 37° to 30°). In all the patients there was a postoperative reduction of the preoperative deformation ranging from 2° to 15°.

Four patients had had to be operated on urgently following the sudden onset, within the last 24 hours, of sphincter impairments and motor deficits in the lower limbs.

The patients were first operated on anterior laterally and subsequently using a posterior approach. In 2 patients only the anterolateral approach was used. In 6 cases (42.8%) the two approaches were undertaken on the same day, whereas in the other cases, and taking into account the clinical condition of the patients as well as the comorbidity, the second operation took place 3-4 days later.

In 10 cases a lateral thoracotomy was performed and in 6 cases a retroperitoneal lumbar approach was used.

The retroperitoneal approach, when possible and taking into consideration the laterality of the paravertebral access, took place from the left. This was due to the presence on the right of the vena cava and because of the greater accessibility to the vertebral field.

For the thoracolumbar option a left side thoracotomy was performed. This was due to the presence on the right of the liver thus making it easier to dislocate the diaphragmatic crura and retract the diaphragmatic cupola downwards.

In all the cases a debridement was done and an expansion mesh was placed.

In the cases operated on using the posterior approach a posterior lateral fusion with an heterologous bone was done.

The suppurating material was always removed. The site was continuously washed using a 0.9% physiological solution and a drainage tube was placed for both types of operations whether using an anterolateral or a posterior approach. The drainage tubes were removed within 24–72 hours.

The patients were immobilized with a corset for about 2 months. An antibiotic therapy was imposed and followed up by colleagues from infectious diseases. It was then suspended on normalisation of the inflammatory markers (white blood cells, ESR, and CRP). The follow-up X-rays took place every 30 days for the first 3 times and then once every six months.

One patient developed a thrombosis to the lower limbs (this was despite the use in all the patients of antithrombosis socks and heparin therapy); the problem resolved itself following further heparin treatment.

All the patients who had shown preoperative deficits underwent physiotherapy in a rehabilitation clinic.

The cage settling ranged from 0 to 3 mm.

In none of the patients was it necessary to reintervene surgically. 

Here, we present two illustrative cases. One of a patient who presented severe preoperative deformities and needed an urgent operation on the lumbar region using a 360° approach. The other of a younger patient who was treated on the dorsal area using the anterior approach. This approach alone was enough to reduce and contain the preoperative rachis deformity. 

## 4. Illustrative Cases

### 4.1. Case 1

This was the case of a 75-year-old male (Case 2, Table 1). Fifteen months earlier, due to the rupture of an abdominal aortic aneurism, this patient had undergone an operation, by intravascular technique, for the placement of an aortic-iliac lumbar prosthesis. For 6 months he had been suffering from lower back pain. The analgesic treatment provided had only eased the pain momentarily. In the last 15 days prior to his operation a progressive weakening of the lower limbs had led to the patient needing a wheel chair. 

Unfortunately, it was in this condition that the patient arrived at the hospital's ER.

**Figure 1:**
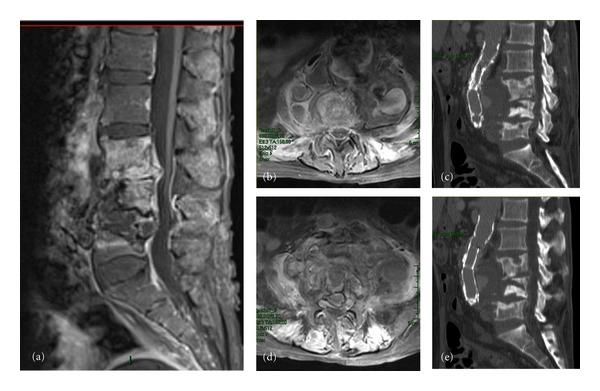
(a) Sagittal MRI, T1 showing the extension of the infectious process to three vertebral bodies L3-L4-L5 and to the intervertebral discs, as in a case of spondylodiscitis. ((b) and (d)) Axial MRI, T2 showing the paravertebral extension of the infection as well as an important compression of the sack and roots by the infection. ((c) and (d)) Sagittal CT showing the erosion and an extended rehashing of the bone as well as the involvement of the aortic prosthesis which has in part been pushed forwards by the infection. The CT also shows the integrity of the vertebral plain of the vertebrae adjacent to the damaged ones.

A CT angiography of the aorta did not show any loss of contrast to the aortic wall while a lumbar-sacral MRI with mdc (Figure 1) revealed a VO involving the vertebral bodies of L3-L4-L5 with invasion of the spinal canal and an important compression of the dural sack.

A colleague from vascular surgery did not attempt the removal of the prosthesis due to the high risk of laceration to the vassal wall.

**Figure 2:**
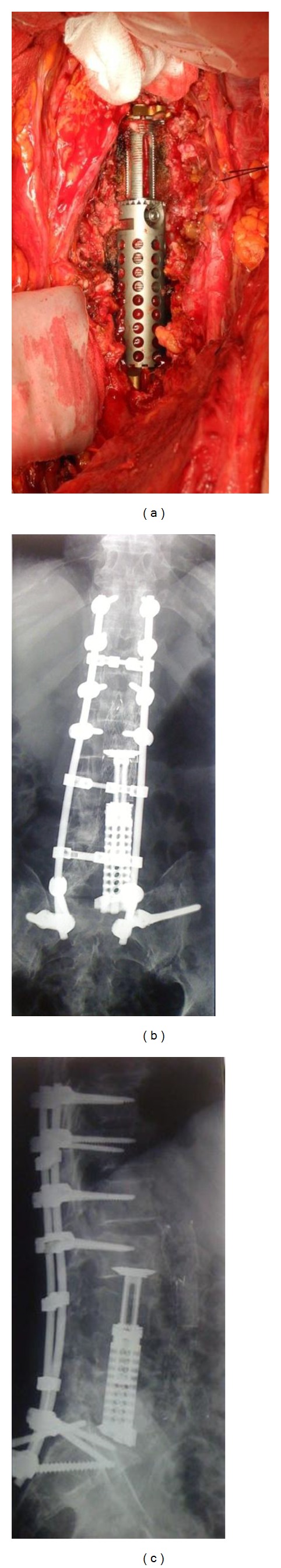
(a) Intraoperative picture showing the placement of the expansion mesh. ((b) and (c)) Follow-up pictures taken 12 months apart. They show the correct placement of the fixation system. The mesh is in place with a 3 mm settling. To be noted here is the natural inclination of the mesh on the inferior end plate. This has allowed for restitution of a partial lordosis to the lumbar-sacral column.

Due to the gravity of his neurological condition (Frankel C) the patient had to be operated on urgently. Under general anaesthesia, with the patient in a lateral position, a left side retroperitoneal lumbar approach was used. The vertebral field was reached by dislocating the aorta, the abdominal viscera, and the urethra medially. A debridement with resection of all infected and necrotic tissues was effected so as to visualise the inferior end plate of L2 and the superior end plate of S1. Samples were taken for histological exams and cultures. An expansion mesh was then placed (Figure 2); it was gradually expanded so as to reduce as much as possible the kyphosis (measurements as to the size of the mesh were based on the preoperative CT). Numerous washes (2000cc) with a 0.9% physiological solution were done and a drainage tube was placed. Then, with the patient in a prone position, the transversal processes were exposed, the pedicle screws were inserted from T11 to the ileum. A decompressive laminectomy of L3, L4, and L5 then followed.

Finally a lateral posterior arthrodesis with autologous and heterologous bone was undertaken and, here too, a drainage tube was placed. The drainage tubes were removed 48 hours after the operation. 

The patient began his physiotherapy and was seated with a corset on the second postoperative day.

The antibiotic treatment was administered intravenously for the first four weeks and then orally for a further 6 months until normalisation of the haematic inflammatory markers.

The first radiological checkup, a CT, took place within 7 days of the operation and was followed by a standard X-ray in two projections. The last checkups, radiological and haematological, 24 months later, have shown no alterations (Figure 2).

### 4.2. Case 2

This was the case of a 34-year-old detainee with back pain which had not been alleviated by pharmacological treatment. The patient was extremely debilitated.

During the neurological exam the patient presented no motor or sensory deficits.

**Figure 3:**
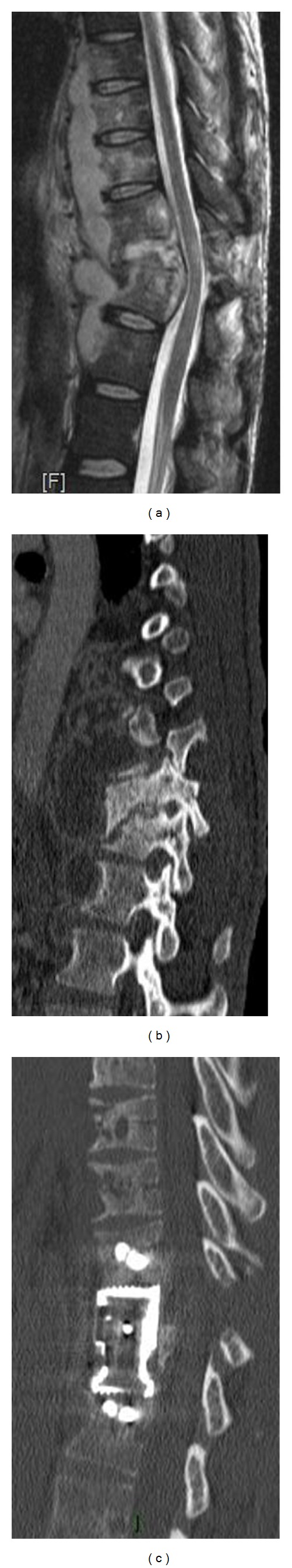
(a) Sagittal MRI T2: here the infectious process has involved two vertebral bodies with an initial tightening of the canal even if it is still possible to identify signs of the liquor around the spinal cord. The paravertebral extension is clearly visible. (b) Sagittal CT: it highlights the marked kyphotic deformation caused by the complete erosion of the bone by the infection, in particular to the inferior vertebral body. (c) Postoperative sagittal CT: highlighted here is the correct positioning of the mesh as well as the restitution of the correct curvature to the dorsal rachises.

He underwent a lumbar MRI with and without contrasts that showed an extended accumulation of suppurating matter in the paravertebral region which involved T11-T12 (Figure 3). This was eroding them and causing the beginning of a kyphosis of the rachis. Initially the patient was treated with the placement of a drainage tube, using the technique of interventionist radiology. The result obtained was unsatisfactory as it failed to reduce pain and it failed to reduce the accumulated suppuration.

**Figure 4:**
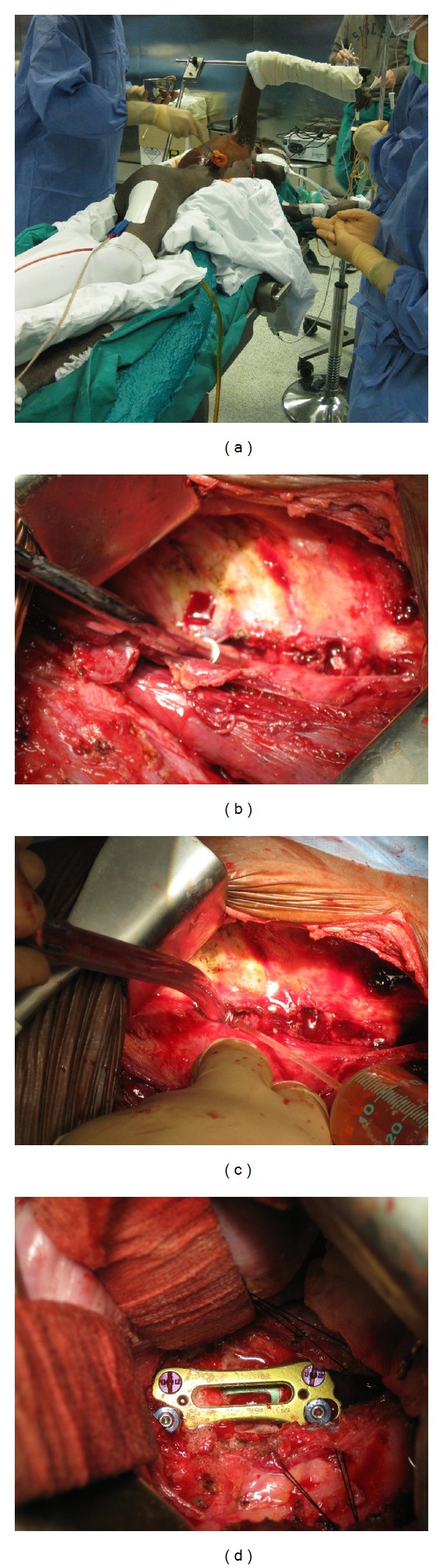
(a) Picture of the position of a patient in the operating room for the lateral thoracotomy approach, with the ipsilateral arm raised. (b) Intraoperative picture showing bone erosion by the infection. (c) During the surgical procedure the site is repeatedly washed with 0.9% physiological solution so as to clear as much as possible the surgical field. (d) Final intraoperative picture. It shows the placement of the mesh and the plaque.

It was then decided that the patient should undergo an operation, a right side lateral thoracotomy, under general anaesthesia. The patient was positioned in lateral decubitus with his right arm raised. An oral-tracheal tube was placed so as to omit the right side lung. The vertebral field was reached by dislocating the lung and the diaphragmatic cupola. There then was a debridement with resection of all infected and necrotic tissue so as to visualise the inferior end plate of T10 and the superior end plate of L1 (Figure 4). 

Samples were collected for the histological and bacteriological exams as well as cultures.

An expansion mesh was placed; it was gradually expanded so as to reduce as much as possible the kyphosis (measurements of the mesh were determined by the preop CT). There then followed numerous washes (2000cc) using a 0.9% physiological solution and a thoracic drain was placed. This latter was removed after about 7 days on complete normalisation of the thoracic region. The patient, wearing a corset, was able to get up 8 days after the operation. 

This patient also had to undergo antibiotic therapy for tuberculosis. The bacteriological exam undertaken during the operation had revealed a tubercular infection. This was confirmed by further cultures. The treatment was carried on for further 6 months.

It was decided that no posterior stabilisation needed to be done considering the age of the patient and the good degree of kyphosis reduction after the initial operation.

In all successive checkups, at the last 36 months, the patient has shown maintenance of the rachises curvature without any signs of kyphosis.

## 5. Discussion

### 5.1. Epidemiology

Spinal infections, also denominated vertebral osteomyelitis (VO), and spondylodiscitis have been known since the end of the XIX century [6, 7]. In the past, in preantibiotic times, they were associated with a high rate of mortality and often were diagnosed postmortem [6, 7].

Spinal infections represent less than 4% of all bone infections [1]. Nowadays the mortality rate has been greatly reduced [4, 8]. However, it remains a challenging pathology to diagnose and treat.

In medical literature the ratio of male to female is 2 M to 1F, which corresponds to our data [9]. The average age of diagnosis in our cases was of 56.6, again in line with the data found in the literature [10].

The majority of patients with spinal infections presented some comorbidity (Table 1): diabetes, debility, drug abuse, and HIV+ [11]. Those patients found positive for the bacillus of Koch often came from countries where tuberculosis was present (Table 1).

The lumbar and dorsal segments were the most frequently affected regions followed in decreasing order by the cervical tract and then the cervicothoracic one [9].

### 5.2. Clinical

The clinical presentation of thoracolumbar VO is generally insidious. It usually begins with back pain, followed later by radicular pains, sensitive/motor deficits, going on to paraparesis and sphincter defects. This gradual escalation of the infectious process is one of the reasons for the late diagnosis [12].

### 5.3. Diagnostic

Laboratory evaluation is fundamental. It included a complete blood cell count, erythrocyte sedimentation rate (ESR), and C-reactive protein (CRP) evaluation, in addition to peripheral blood cultures [13]. These analyses were used to evaluate the efficiency of the medical and surgical treatments. Their normalisation was necessary to suspend antibiotic treatment.

The radiological exams included standing plain radiographs, MRI with gadolinium and CT. The first one at the beginning of the pathology can come up negative or be difficult to interpret. The MRI with gadolinium, is the optimal exam as it can show bone and disc involvement, the presence of paravertebral accumulations and epidural abscesses, spinal cord compression, and any possible ischemic lesions [14, 15]. The CT is needed to program the surgical intervention and for the placement of the prosthesis as the information obtained regards the density and erosion of the bone.

The percentage of positive results from the computed tomography-guided needle biopsy is between 29%, 51%, and 96% [5, 8, 9]. Also the percentage of microbiological diagnosis following an intraoperative biopsy has a high degree of variability in literature 80%, 18%, and 59%; [8–10] in our case studies the cultures were 56% positive.

This was due to the fact that a lot of patients came to the surgeon's attention following the onset of neurological problems and after having already begun an antibiotic therapy.

81% (13 cases) of the patients came to our department after having seen other specialists in the field and after having been administered antibiotics for a number of weeks.

### 5.4. Treatment

To date there are no guidelines regarding medical treatment associated or not with surgical treatment. Moreover, with regard to surgical treatment, there is no agreement on whether to follow only a debridement or to position an allograft strut grafting, or to use a prosthesis in different materials (stainless steel and titanium) [16, 17]. Different surgical approaches may be used anteriorly, posteriorly, or both.

Traditional medical treatment calls for the administration of intravenous antibiotics for 4 weeks, then orally (3–12 months) until normalisation of the inflammatory markers associated with the positioning of a corset [1].

As stated above there is, as yet, no agreement on how or when to intervene surgically [5]. In our institute, we maintain, along with some authors, that surgical treatment is indicated in failure of nonoperative treatment (persistent pain, persistent abnormal ESR e CRP, and persistent or increase of the abscess in MRI), extensive bone destruction, kyphosis, and neurological deterioration (motor deficits, paraparesis or paraplegia, and bladder dysfunction). The policy in our institute is to intervene surgically within 24 hours on patients who are deteriorating neurologically.

In this study 75% (13) of the patients had preoperative motor deficits. But, whatever the case, surgical treatment must always be associated with the administration of antibiotics.

A few years ago the use of spinal instrumentation in VO was a much discussed topic [18–20]. Now many papers on the use of anterior stabilisation with debridement associated with or without posterior stabilisation have been published [16, 17].

The advantages of this technique would be the reduction of vertebral deformities, the reduction of pain, an earlier patient mobilization, and the decompression of neural structures.

Few studies have been undertaken on which materials to use. But it would seem that the adherence and persistence of bacteria are higher with stainless steel devices than with titanium [17]. In addition to this, biofilms tend to form more frequently on stainless steel than on titanium [21, 22].

In our case studies 16 patients were operated on using an anterolateral approach. It was not always possible to perform both the anterior and posterior approach on the same day due to the clinical condition and comorbidity of the patient.

In approaches to the dorsal rachises it is very important to do, when possible, a spinal angiography so as to know the origin of the artery of Adamkiewicz. This serves to avoid damaging it and to reduce vascularization of the spinal cord in patients who often already have a neurological damage. It also serves to link the intercostal artery, if necessary, after the origin of the artery itself. 

In our cases we found no relevant complications nor any need to reintervene surgically. 

The inferior and superior sides of the mesh should be placed on vertebral end plates which are not affected by the infection and not on the cancellous bone even if this latter is healthy, as some authors show [5, 17]. This is so as to avoid excessive settling, in our cases this was from 0 to 3 mm.

In the majority of works, regarding treatment with anterior surgery, there have been published accounts of cases of VO involving one or two adjacent vertebral bodies and their discs.

In these papers 2 cases were described, one lumbar and one thoracic, of triple corpectomy for an infection which involved three vertebral bodies. In both cases the spinal infection was secondary to an adjacent infected aortic graft. In our cases we did not remove any vascular prosthesis as they had been in place for over a year and the risk of damage to the vassal wall was too high. However, these patients followed an antibiotic therapy of 9 months and successive checkups showed that the haematic inflammatory markers were within the norm.

One of the first authors to talk about spinal fusion by anterior approach for patients with spinal infections caused by the BK was Hodgson in 1960 [23]. The anterolateral thoracotomy or retroperitoneal approach allows direct access to the vertebrae and thus to the infection which helps to reduce more efficiently the vertebral deformities. In our cases it was decided to perform a posterior approach as well when it was necessary to reduce a significant kyphosis; in obese patients, an important spinal cord compression was present which necessitated decompression both anteriorly and posteriorly by laminectomy.

The anterior and posterior approach would normally be followed in the same operation [16] to reduce the risk of infection, to reduce blood risk, to reduce the length of hospitalisation, to allow for precocious mobilization, and so as to avoid that the patient undergoes two anaesthesias.

Unfortunately this may not always be possible as certain patients present comorbidity and are medically unstable. In our case studies, whether the patients had one or two operations, the outcome was always good.

## 6. Conclusion

Spinal infections are insidious pathologies which can lead to motor and sphincter damages. In their early stages they can be difficult to diagnose if an MRI with gadolinium is not done, as standard radiography results can come up negative.

The anterolateral surgical treatment with placement of a device associated or not with a posterior stabilisation is indicated for patients unresponsive to traditional treatments, with a significant deformation of the rachis which necessitates 360° stabilisation, in the presence of neurological damage which requires decompression of the spinal cord or the roots either by an anterior or a posterior approach. In fact, surgical treatment may even become urgent if there has been significant neurological deterioration in the last 24 hours. The anterolateral approach allows access to all the vertebral bodies involved in the infection so as to follow a complete debridement, especially in extended infections, and to place a prosthesis in the absence of complication and with a rapid clinical improvement for the patient.

The expandable titanium mesh cage allows through gradual distension to reduce important vertebral deformities with the effective absence of risk of infection to the prosthesis itself.

The retrospective analysis of our cases leads to the conclusion that there are two principal objectives to be reached:

1. an extended drainage of the area affected by the infective process;
2. a reduction of the rachises deformation, if present, and a stabilisation of the vertebral column.

Both these objectives are reachable by using a combined posterior and anterior approach.

## Figures and Tables

**Table 1:** This table shows general data regarding our patients including sex, age, origin, and presence of other pathology that can favourite the onset of infection. In the table are also indicated the affected levels, the responsible microorganisms, and the level treated whit stabilization. There are also summary data regarding pre-operative and post-operative neurological status valuated with Frankel Scale (*Grade A*: complete neurological injury—no motor or sensory function clinically detected below the level of the injury. *Grade B*: preserved sensation only—no motor function clinically detected below the level of the injury; sensory function remains below the level of the injury but may include only partial function (sacral sparing qualifies as preserved sensation). *Grade C*: preserved motor nonfunctional—some motor function observed below the level of the injury, but is of no practical use to the patient. *Grade D*: preserved motor function—useful motor function below the level of the injury; patient can move lower limbs and walk with or without aid, but does not have a normal gait or strength in all motor groups. *Grade E*: normal motor—no clinically detected abnormality in motor or sensory function with normal sphincter function; abnormal reflexes and subjective sensory abnormalities may be present. There are also summary data regarding duration of symptoms and duration of followup.

Patients	Sex	Age	Birth place	Comorbidity	Levels of infection	Organisms	Instrumented levels	Neurological status preop/postop	Followup	Duration of symptoms
1	M	56	Italy	Obesity, thoracic aortic prosthesis	T7–T9	Culture negative	T4–T12	C/D	18 months	2 weeks
2	M	75	Italy	Lumbar aorto-iliac prosthesis	L3–L5	*Staphylococcus aureus *	T11-ileum (sacro-iliac screw)	C/D	24 months	12 weeks
3	F	80	Italy	Diabetes mellitus	T7-T8	*Staphylococcus aureus *	T5–T10	E/E	33 months	4 weeks
4	M	61	Italy	Drug abuser	T8-T9	Culture negative	T6–T11	C/C	24 months	10 weeks
5	M	21	Somalia	Debility	T9	BK	T7–T11	E/E	48 months	16 weeks
6	M	34	Ethiopia	Debility	T11-T12	BK	Only anterior approach	D/E	36 months	12 weeks
7	M	35	Italy	Drug abuser	T9-T10	BK	T7–T12	C/E	23 months	3 weeks
8	F	64	Peru	None	L2-L3	*Staphylococcus aureus *	D11-S1 (spondilolistesi L5-S1)	E/E	40 months	12 weeks
9	M	59	Ethiopia	Diabetes mellitus	T7–T9	Culture negative	T5–T11	D/E	37 months	6 weeks
10	F	47	Kenya	Hiv+	T8-T9	*Staphylococcus epidermidis *	T6–T11	C/D	25 months	10 weeks
11	F	53	Italy	Thoracic aortic prosthesis	T11-T12	Culture negative	T9-L2	C/C	42 months	2 weeks
12	M	65	Somalia	Debility	T9-T10	Culture negative	T8–T11	D/E	30 months	8 weeks
13	M	48	Italy	None	L3-L4	*Staphylococcus aureus *	Only anterior approach	E/E	45 months	16 weeks
14	M	57	Romania	Alcohol abuser	L2-L3	Culture negative	T12-L5	A/A	28 months	1 week
15	F	69	Romania	Drug abuser	L3-L4	Culture negative	L1-S1	C/E	28 months	3 weeks
16	F	80	Italy	Diabetes mellitus	L2-L3	*Staphylococcus epidermidis *	T12-L5	D/E	44 months	12 weeks
